# *Metashinobius* gen. nov., the second Asian genus with uncleared subfamily placement of Trechaleidae (Araneae, Lycosoidea) from southwest China, with the description of a new species

**DOI:** 10.3897/BDJ.13.e153601

**Published:** 2025-06-27

**Authors:** Yufan Cheng, Jie Liu, Mian Wei

**Affiliations:** 1 The Arachnid Rescorce Centre of Hubei Province & Centre for Behavioral Ecology & Evolution, School of Life Sciences, Hubei University, Wuhan, China The Arachnid Rescorce Centre of Hubei Province & Centre for Behavioral Ecology & Evolution, School of Life Sciences, Hubei University Wuhan China; 2 The State Key Laboratory of Biocatalysis and Enzyme Engineering of China, School of Life Sciences, Hubei University, Wuhan, China The State Key Laboratory of Biocatalysis and Enzyme Engineering of China, School of Life Sciences, Hubei University Wuhan China; 3 Hubeiate Key Laboratory of Regional Development and Environmental Response, Faculty of Resources and Environmental Science, Hubei University, Wuhan, China Hubeiate Key Laboratory of Regional Development and Environmental Response, Faculty of Resources and Environmental Science, Hubei University Wuhan China

**Keywords:** Asia, biodiversity, morphology, new genus, taxonomy

## Abstract

**Background:**

Trechaleidae Simon, 1890 is a relatively small spider family comprising 17 genera and 136 species, most of which are distributed in South America and only one genus and three species are distributed in Asia; however, these have never been reported in southwest China.

**New information:**

A new genus, *Metashinobius* **gen. nov.**, is established, based on a new species from Guizhou Province, southwest China: *Metashinobiushikariae* **sp. nov.** (♂). This is the second Asian genus of Trechaleidae. Diagnosis, detailed descriptions, colour illustrations and distribution map of the new species are provided. The type specimen of the new species is deposited in the Centre for Behavioural Ecology and Evolution (CBEE), Hubei University, Wuhan, China.

## Introduction

Trechaleidae Simon, 1890 is a relatively small spider family including 17 genera and 136 species, amongst which 16 genera and 133 species are distributed in South America and only one genus, *Shinobius*, is distributed in Japan (World Spider Catalog 2025). The genus *Shinobius* was established by Yaginuma in 1991, with *Cispiusorientalis* Yaginuma, 1967 as the type species. According to the low cephalic region, the presence of the ventrodistal rim of male palpal tibia and other six characteristics mentioned in his article, *Shinobius* was placed in Rhoicininae Exline, 1950, Pisauridae Simon, 1890 (Yaginuma 1991). Since the subfamily Rhoicininae was transferred to Trechaleidae (Sierwald 1993, Griswold 1993), *Shinobius* became the first Asian trechaleids genus. In recent years, Chinese researchers reported two additional *Shinobius* species from south Xizang, China (Zhang et al. 2024, Wang et al. 2024), which indicated that the diversity of Asian trechaleids had been underestimated.

During the examination of the specimens collected from southwest China, an unknown species resembling *Shinobius* in somatic characteristics and genitalic structures , such as the anterior eye row being distinctly wider than the posterior median eye row, the basal part of the cymbial retrolateral margin being membranous, the presence of the large median apophysis and the membranous conductor, was discovered, but the presence of a modified retrolateral tibial apophysis indicates that it is reasonable to establish a new genus to accommodate the new species. Therefore, we report the new discovery in this study, by providing the detailed descriptions, colour illustrations and distribution map.

## Materials and methods

All specimens were preserved in 75% ethanol and examined using an Olympus SZX7 stereomicroscope. Male palps were dissected from the spider bodies for examination and photography. Epigynes were cleared with Proteinase K to reveal their internal structures. Photographs were captured with a Canon EOS 90D wide-zoom digital camera (8.5 megapixels) mounted on an Olympus BX43 compound microscope and the images were stacked and processed using Helicon Focus 7.0.2 image stacking software. The map was generated by SimpleMappr (https://www.simplemappr.net). Leg measurements are provided as total length, including segments (femur, patella, tibia, metatarsus, tarsus). Measurements were taken exclusively from the left structures (e.g. pedipalps, legs) and all measurements are in millimetres. The holotype has been deposited in the Centre for Behavioural Ecology and Evolution, College of Life Sciences, Hubei University, Wuhan, China (CBEE). Abbreviations are as follows:

Morphological charactristics: ALE = anterior lateral eye; AME = anterior median eye; AME–ALE = distance between AME and ALE; AME–AME = distance between AME and AME; ALE–PLE = distance between ALE and PLE; AME–PME = distance between AME and PME; Fe = femur; Mt = metatarsus; PLE = posterior lateral eye; PME = posterior median eye; PME–PLE = distance between PME and PLE; PME–PME = distance between PME and PME; Ti = tibia. Spination: d = dorsal; p = prolateral; pv = prolateral ventral; r = retrolateral; rv = retrolateral ventral; v = ventral.

## Taxon treatments

### Metashinobius

Wei & Liu gen. nov.

738D740C-B5D5-5160-9C0B-D6238BC7B7D7

074AF347-9156-44E7-B171-C00A11A0F9CB

Metashinobius hikariae Wei & Liu. Type species.

#### Description

Male small-sized (total length approximately 5.00). In dorsal view, carapace pear-shaped, anterior eye row slightly recurved, ALE subequal to AME; posterior eye row recurved, posterior eyes larger than anterior eyes and anterior eye row wider than posterior median eye row. Secondary eyes with grate-shaped tapetum. Cheliceral promargin with 3 teeth, retromargin with 4 teeth, with the third one smaller than the others. Leg formula: 1423. Trochanters notched. Sternum heart-shaped. Colulus present. Colour in alcohol: carapace blackish-green, with a large central pattern and dark margin, four stripes present behind each posterior eyes; cephalic groove and radial furrow black; fovea distinct, longitudinal. Legs yellow-brown, femurs, patellae and the distal tip of tibiae with dark patterns, metatarsi and tarsi brownish-red. Each paired tarsal claw with 12 teeth, unpaired claw with one tooth. For leg spination, see in species description. Abdomen with dark patterns. Male palp same as for the species.

Females unknown.

#### Diagnosis

**Figure 1.**
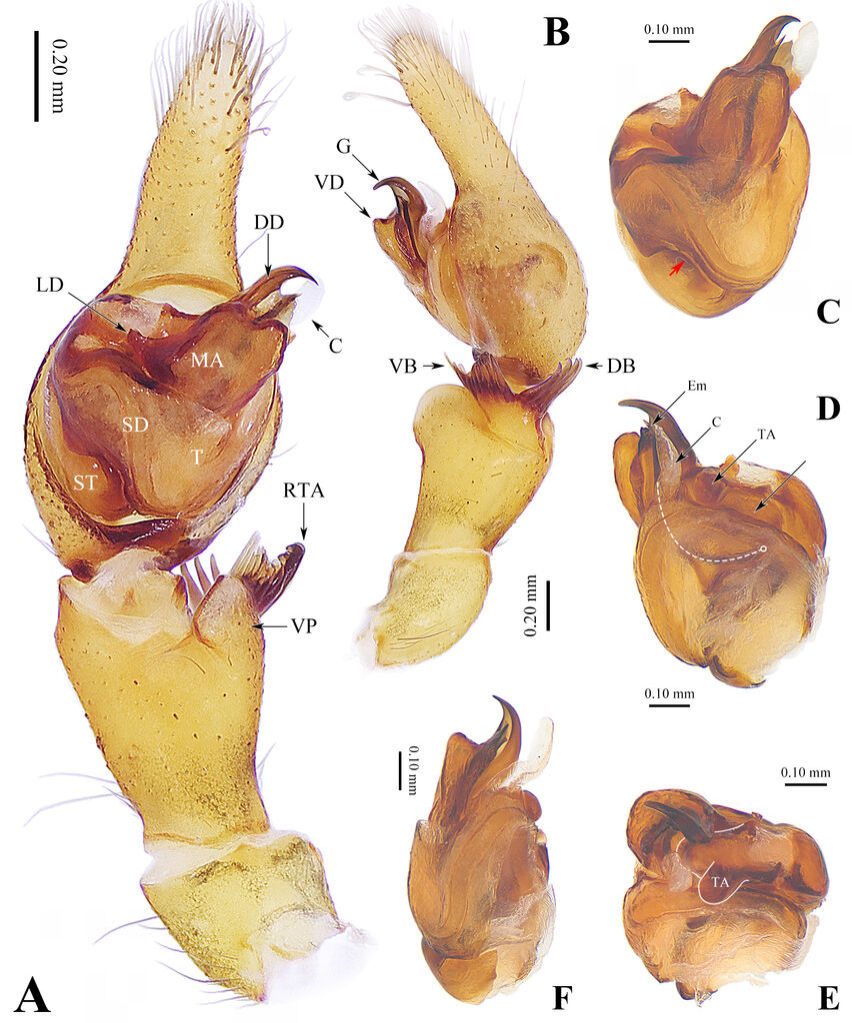
*Metashinobiushikariae* sp. nov. **A**, **B** left palp; **C**–**F** right palpal bulb. **A, C** ventral view; **B, F** retrolateral view; **D** dorsal view; **E** apical view. The red arrow in **C** indicates the tegular probasal notch, the white dashed line in **D** indicates the hidden embolus. Abbreviations: C = conductor; DB = dorsal branch of retrolateral tibial apophysis; DD = dorsal division of median apophysis; Em = embolus; G = guide groove; LD = lateral division of median apophysis; MA = median apophysis; RTA = retrolateral tibial apophysis; SD = sperm duct; ST = subtegulum; T = tegulum; TA = terminal apophysis; VB = ventral branch of retrolateral tibial apophysis; VD = ventral division of median apophysis; VP = ventrodistal protuberance.

**Figure 2.**
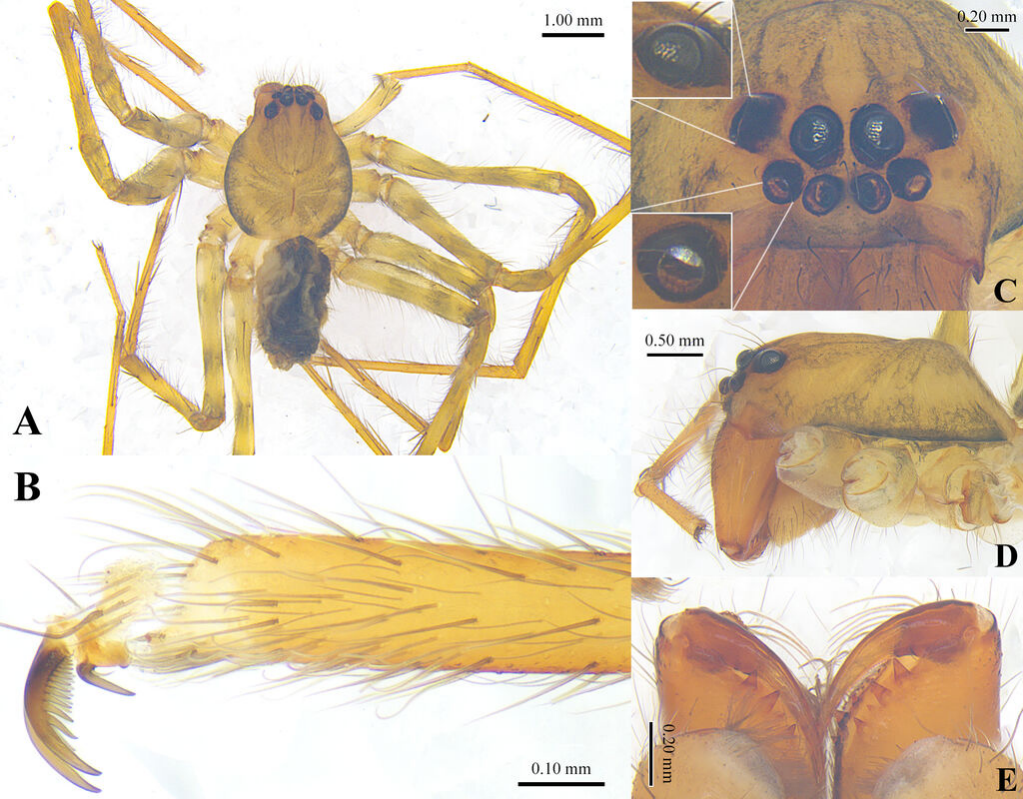
Male holotype of *Metashinobiushikariae* sp. nov. **A** habitus, dorsal view; **B** the fourth left tarsal claw, retrolateral view; **C** ocular area, frontal; **D** carapace, lateral view; **E** chelicerae, ventral view.

The new genus resembles *Shinobius* Yaginuma, 1991 and can be distinguished from the latter by: 1) having 4 postmarginal teeth (Fig. 2E), versus having 3 postmarginal teeth in the latter (Sierwald 1993, Wang et al. 2024, Zhang et al. 2024); 2) the presence of a modified retrolateral tibial apophysis and a large ventrodistal protuberance (Fig. 1A and B), versus lacking the retrolateral tibial apophysis and having a small ventrodistal protuberance in the latter (figs. 20 and 21 in Sierwald (1993); figs. 3A and B in Wang et al. (2024); figs. 2A and B in Zhang et al. (2024)); 3) the median apophysis is thick, with a uplifted ventral division and a lateral division and the tip of the guide groove points downwards (Fig. 1A, C and D), versus being thin, with a flat ventral division, without lateral division and the guide groove pointed upwards (figs. 20 and 21 in Sierwald (1993); figs. 1A–D in Wang et al. (2024); figs. 2A, B, 3A and C in Zhang et al. (2024)).

#### Etymology

The generic name is derived from its similarity to *Shinobius* and the Greek adjective Meta for after. The gender is masculine.

#### Distribution

Guizhou Province, China

#### Notes

We place this new genus under the family Trechaleidae by: 1) the posterior eye row being recurved; 2) the presence of a ventrodistal rim of the male palpal tibia; 3) the median apophysis being large and divided into ventral and dorsal divisions, with a dorsal guide groove located on the dorsal division (Carico 1993). The new genus resembles *Shinobius* most, but cannot be placed in the subfamily Rhoicininae as the latter, due to the presence of the unique retrolateral tibial apophysis (which is absent in all genera of Rhoicininae); nor can it be accommodated within other trechaleids clade such as subfamily Trechaleinae Simon 1890, based on the Asian distribution and the relatively long anterior eye row. Definitive subfamily classification requires additional specimens to supplement critical morphological data; therefore, the subfamily placement is withheld in the current paper.

### Metashinobius hikariae

Wei & Liu sp. nov.

AF82998F-B91D-5FE1-BE7E-E3499E5487C7

51B8C4AF-7E19-46C8-8095-E00A8BA2CCD8

#### Materials

- **Type status:** Holotype. **Occurrence:** recordedBy: Mian Wei; occurrenceID: 7C0E161A-8E0E-5202-BE4F-62BFF4BAF049; **Location:** country: China; stateProvince: Guizhou; locality: Qiandongnan Miao and Dong autonomous prefecture, Leishan County, Xiannutang; verbatimElevation: 1712; verbatimCoordinates: 26.3748°N, 108.2044°E; **Event:** eventDate: 29 Jul 2021

#### Description

**Male** (holotype). Habitus as in Fig. 2. Total length 4.58. Carapace 2.46 long, 2.17 wide. Abdomen 2.12 long, 1.22 wide. Eye diameters and interdistances: AME 0.15, ALE 0.12, PME 0.17, PLE 0.20; AME–AME 0.06, AME–ALE 0.06, PME–PME 0.11, PME–PLE 0.15, AME–PME 0.10, ALE–PLE 0.20. Measurements of legs: I 13.02 (3.52, 1.05, 3.85, 3.49, 1.44), II 12.75 (3.28, 0.90, 3.76, 3.31, 1.50), III 10.66 (2.87, 0.86, 2.97, 2.90, 1.06), IV 12.29 (3.32, 0.92, 3.22, 3.49, 1.34). Leg spination: I: Fe d2 p2 r2, Ti p2 r2 pv3 rv3, Mt p3 r3 pv3 rv3; II: d2 p2 r2, Ti p2 r2 pv3 rv3, Mt p3 r3 pv3 rv3 v1; III: Fe d3 p2 r2, Ti d3 p2 r2 pv3 rv3, Mt p3 r3 pv3 rv3 v1; IV: Fe d2 p2 r1, Ti d2 p2 r2 pv2 rv2, Mt p3 r3 pv3 rv3 v1. Chelicerea with 3 promarginal teeth and 4 retromarginal teeth, the third retromarginal teeth smallest.

Palp (Fig. 1). Retrolateral tibial apophysis strong, bloom-shaped, consisting of a root and a crown, the latter consisting of 16 spine-shaped projects and divided into two branches, ventral branch fishtail-shaped and consisting of 12 projects and dorsal branch consisting of 4 projects. Ventrodistal protuberance large and distinct. Cymbium with relatively short distal part and sclerotised ecto-basal part and with membranous basal part of cymbial retrolateral margin. Tegulum large, with tegular probasal notch. Sperm duct wavy. Median apophysis large and strongly sclerotised, divided into three divisions; lateral division thin and digitate; ventral division broad and uplifted, approximately rectangular; dorsal division with a claw-shaped guide groove pointed downwards and a laminar margin. Conductor long and membranous. Terminal apophysis semicircular. Embolus long and arc-shaped, hidden by median apophysis from ventral view.

**Female.** Unknown.

#### Diagnosis

The males of *Metashinobiushikariae* sp. nov. resembles *Shinobius* species, particularly *S.orientalis* (Yagunima, 1967) by: 1) the relatively small subtegulum, which is obviously smaller than the tegulum from ventral view; 2) having a concavity on the posterior margin of tegulum; 3) the wavy sperm duct; 4) the irregular-shaped median apophysis (A; fig. 20 in Sierwald (1993); fig. 2n in Serita (2021)). In the other two *Shinobius* species, the subtegulum is larger than or subequal to the tegulum from ventral view, the posterior margin of tegulum is straight, the sperm duct is approximately straight from dorsal view and the median apophysis is horizontally flipped “9”-shaped (fig. 3A in Wang et al. (2024); fig. 2A in Zhang et al. (2024)). However, the new species can be distinguished from *S.orientalis* by: 1) have 4 postmarginal teeth (Fig. 2E), versus having 3 in the latter; 2) having a modified retrolateral tibial apophysis and a larger ventrodistal protuberance (Figs. 1A and B), versus lacking the retrolateral tibial apophysis and having a smaller ventrodistal protuberance in the latter (figs. 20 and 21 in Sierwald (1993); fig. 2n in Serita (2021)); 3) the median apophysis being larger than the subtegulum from ventral view and the distal tip of the guide groove pointing downwards (Fig. 1A), versus being smaller than the subtegulum and with the distal tip of the guide groove pointing upwards in the latter (figs. 20 and 21 in Sierwald (1993)).

#### Etymology

The specific epithet is from “Hikari Kagura”, one of the main protagonists from the animation “Shoujo Kageki Revue Starlight”, whose weapon bloomed at the crucial moment, referring to the bloom-shaped retrolateral tibial apophysis of the male palp of the new species.

#### Distribution

**Figure 3.**
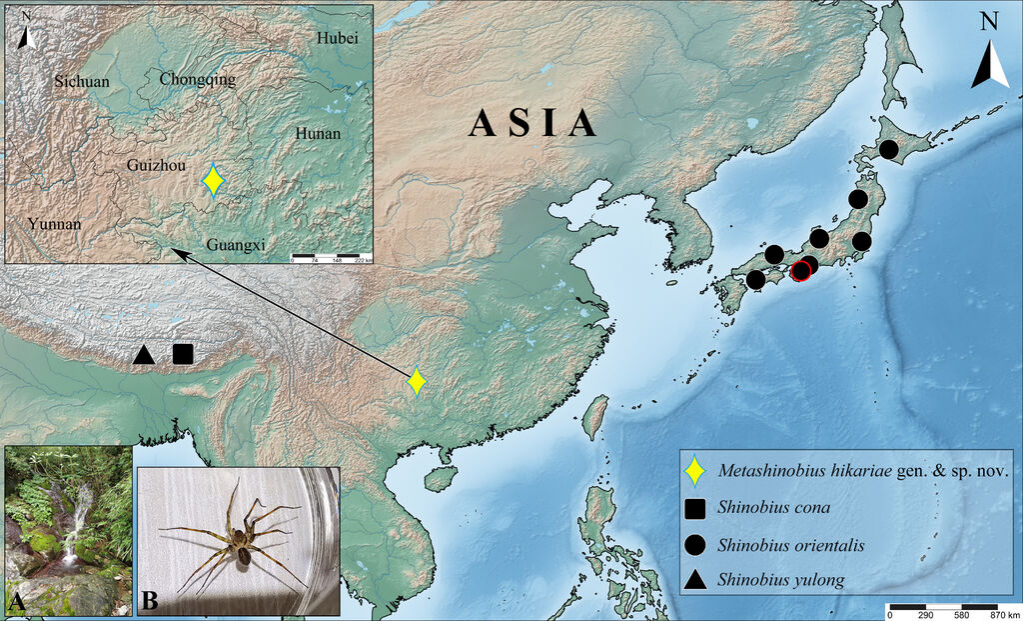
Distributions of *Metashinobiushikariae* sp. nov. and *Shinobius* spp. **A** type locality; **B** living male. The circle with red border indicates the type locality of *S.orientalis*.

Guizhou Province, China (only known from the type locality) (Fig. 3).

## Supplementary Material

XML Treatment for Metashinobius

XML Treatment for Metashinobius hikariae
